# Prolonged corrected QT interval is associated with cardiac sympathetic nervous function overactivity in patients with severe aortic stenosis: assessment by 123I-metaiodobenzylguanidine myocardial scintigraphy

**DOI:** 10.1007/s00380-025-02550-6

**Published:** 2025-05-11

**Authors:** Yukihiro Fukuda, Yoshifumi Nishio, Hironori Miyazaki, Yoshiyuki Okada, Hironori Ueda, Shinya Takahashi, Yukiko Nakano

**Affiliations:** 1 Department of Cardiovascular Medicine, Hiroshima Prefectural Hospital, 1-5-54, Ujina-kanda, Minami-ku, Hiroshima, 7348530 Japan; 2 Department of Special Care Dentistry, Hiroshima University, Hiroshima, Japan; 3 Department of Cardiovascular Surgery, Hiroshima University Graduate School of Biomedical and Health Sciences, Hiroshima, Japan; 4 Department of Cardiovascular Medicine, Hiroshima University Graduate School of Biomedical and Health Sciences, Hiroshima, Japan

**Keywords:** Aortic stenosis, Electrocardiogram, QTc prolongation, Cardiac autonomic system

## Abstract

Prolonged corrected QT interval (QTc) is known to be associated with adverse cardiovascular events in patients with heart failure. The delayed heart-to-mediastinum (*H*/*M*) ratio obtained from 123I-metaiodobenzylguanidine (MIBG) myocardial scintigraphy is a marker of cardiac sympathetic nervous (CSN) activity and has been proposed as a prognostic marker of severe aortic stenosis (AS). However, the association between prolonged QTc and CSN overactivity in patients with AS remains unclear. This study retrospectively analyzed 83 patients with severe AS who underwent electrocardiography, echocardiography, and 123I-MIBG scintigraphy. Prolonged QTc was defined as QTc > 450 and > 470 ms in men and women, respectively. CSN overactivity was defined as delayed *H*/*M* ratio < 2.2 and washout rate (WR) > 34%. Prolonged QTc was detected in 14 patients, and these patients had higher left ventricular (LV) mass index and lower LV ejection fraction as compared to those with normal QTc. A significantly higher proportion of patients with prolonged QTc demonstrated CSN overactivity (*p* = 0.02). In addition, the prolonged QTc group had a lower delayed *H*/*M* ratio and higher WR. QTc was inversely correlated with the delayed *H*/*M* ratio in men (*r* =  − 0.53, *p* = 0.02) and women (*r* =  − 0.29, *p* = 0.02). QTc was positively correlated with WR in men (*r* = 0.55, *p* = 0.01) and women (*r* = 0.42, *p* = 0.001). Multivariate analysis identified age and prolonged QTc as significantly associated with CSN overactivity. Thus, prolonged QTc is associated with CSN overactivity, as assessed using 123I-MIBG scintigraphy in patients with severe AS.

**Supplementary Information:**

The online version contains supplementary material available at 10.1007/s00380-025-02550-6.

## Introduction

Aortic stenosis (AS) is a common valvular disease that causes left ventricular (LV) outflow obstruction and is associated with dismal outcomes, such as heart failure and sudden death [1, 2]. In addition, AS has been linked to heightened systemic sympathetic nervous activity as evidenced by increased muscle sympathetic nerve activity [3]. In clinical practice, 123I-metaiodobenzylguanidine (MIBG) myocardial scintigraphy has been widely used to assess cardiac sympathetic nervous (CSN) activity [4–6]. Previous studies have shown that a reduced delayed heart-to-mediastinum (*H*/*M*) ratio and an increased washout rate (WR), representing CSN overactivity, are associated with poor prognosis in patients with severe AS [7, 8].

Prolonged corrected QT interval (QTc) has been associated with increased cardiac sympathetic tone in patients with hypertension, collagen diseases, and even in healthy individuals [9–12]. Furthermore, prolonged QTc is considered a marker of advanced disease stage, linked to impaired LV function and increased long-term mortality following aortic valve replacement [13, 14]. However, no studies have investigated whether QTc is associated with CSN activity in patients with severe AS. Therefore, this study sought to examine whether prolonged QTc is associated with CSN overactivity in patients with severe AS.

## Materials and methods

### Study design and population

Between January 2015 and April 2020, a total of 248 patients with known or suspected severe AS were admitted to our hospital to assess indication for transcatheter aortic valve replacement (TAVR) or surgical aortic valve replacement. Severe AS was defined as (1) an aortic valve area (AVA) of < 1.0 cm^2^ (or AVA indexed by body surface area < 0.65 cm^2^/m^2^) or (2) a resting or inducible peak trans-aortic velocity > 4.0 m/s or (3) a resting or inducible mean pressure gradient of > 40 mmHg [15]. Of the 248 patients, 109 underwent 123I-MIBG scintigraphy for heart failure evaluation.

**Fig. 1:**
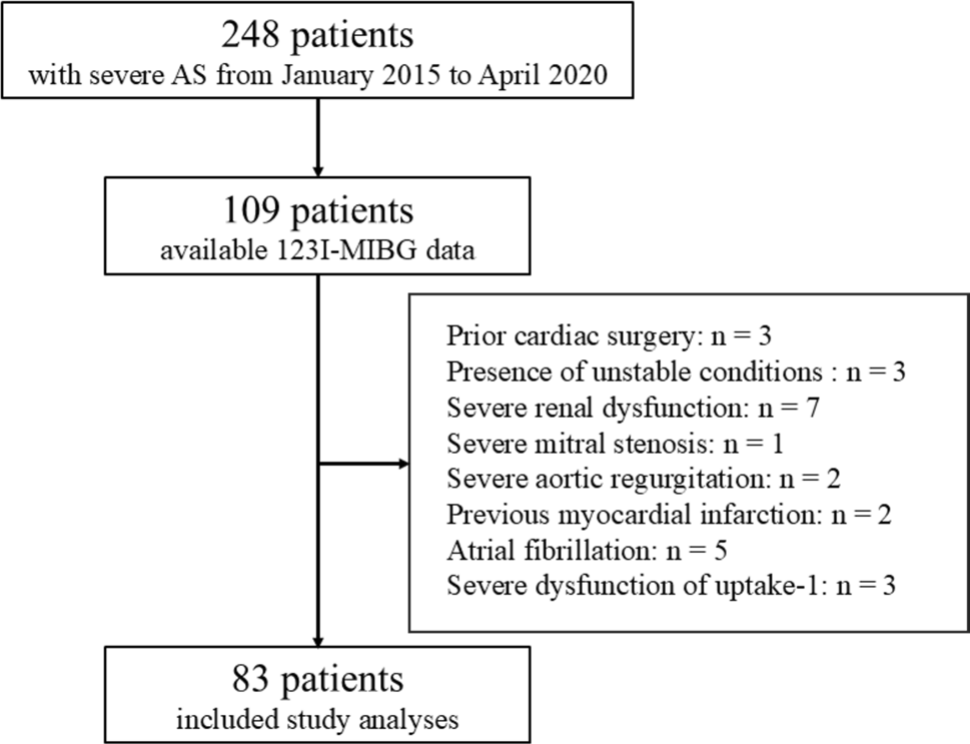
Study flowchart. *MIBG* metaiodobenzylguanidine

The exclusion criteria were as follows: (1) prior cardiac surgery (*n* = 3), (2) presence of unstable conditions (*n* = 3), (3) severe renal dysfunction (estimated glomerular filtration rate < 30 mL/min/1.73 m^2^) (*n* = 7), (4) severe mitral stenosis (*n* = 1) or severe aortic regurgitation (*n* = 2), (5) previous myocardial infarction (*n* = 2), (6) atrial fibrillation (*n* = 5), and (7) severe dysfunction of uptake-1 (early *H*/*M* ratio < 2.2) (*n* = 3) [16]. After applying the exclusion criteria, 83 patients with severe AS were included in the final analysis (Fig. 1). No patients had Parkinson’s disease or were taking medications known to interact with 123I-MIBG, such as amiodarone, labetalol, reserpine, tricyclic antidepressants, sympathomimetic amines, and serotonin–norepinephrine reuptake inhibitors [17].

The retrospective study protocol was approved by the Institutional Ethics Committee of Hiroshima University Hospital, and written informed consent was obtained from all participants.

### Electrocardiogram

A standard 12-lead electrocardiogram (ECG) was performed for all patients. A diagnostic information system (PRM-4000; Nihon Kohden, Tokyo, Japan) automatically measured the QT intervals. The corrected QTc was calculated using Bazett formula: QTc = QT divided by the square root of the RR interval. A prolonged QTc was defined as > 450 and > 470 ms in men and women, respectively [14].

### Transthoracic echocardiography

Transthoracic echocardiography evaluations were performed by three experienced cardiac sonographers who were blinded to the clinical status of the patients. Examinations were conducted using a Vivid E9 ultrasound system equipped with a 2.5-MHz transducer (GE Vingmed Ultrasound, Horten, Norway) within one week prior to 123I-MIBG scintigraphy. All imaging data were digitized and stored on optical disks for offline analysis using EchoPac software version 112 (GE Vingmed Ultrasound) [18]. The echocardiographic parameters were measured according to the recommendations of the American Society of Echocardiography [19, 20]. The AVA and mean pressure gradient were calculated using the continuity equation and Bernoulli’s formula, respectively. LV ejection fraction (LVEF) was assessed using the biplane Simpson formula, and the LV internal dimension (LVID), interventricular septal thickness (IVS), and posterior wall thickness (PWT) were measured at the end of diastole. The LV mass was calculated using the following formula: LV mass (g) = 0.8 × 1.04 [(LVID + IVS + PWT)^3^ − (LVID)^3^] + 0.6. The LV mass index (LVMI) was calculated as the LV mass divided by the body surface area.

### Cardiac 123I-MIBG scintigraphy

All patients underwent 123I-MIBG scintigraphy using a dual-detector 90° γ-camera (Brightview XCT, Philips Healthcare, Milpitas, CA, USA) equipped with a medium-energy general collimator. 123I-MIBG (Fujifilm RI Pharma Co., Tokyo, Japan) was administered intravenously at a dose of 111 MBq. Anterior planar images were captured at 15 (early image) and 210 (delayed image) min after 123I-MIBG injection [21, 22]. The imaging parameters were as follows: image acquisition time, 5 min; matrix size, 128 × 128; magnification factor, 1.46; energy window, 159 keV with a 20% energy window; pixel size, 3.2 mm. Images were analyzed based on the region of interest determined using dedicated software (Jetpack, Hitachi, Japan) by an experienced radiology technician who was blinded to the clinical status of the patients. The Jetpack software enabled semiautomated determination of the *H*/*M* ratios and standardization for medium-energy collimator conditions. Early and delayed *H*/*M* ratios were calculated by measuring the average counts in each region on the anterior views of the planar images [21–24]. The WR was calculated as follows: [(*H − M*) early* − *(*H − M*) delayed/*k*] × 100/(*H − M*) early with background subtraction and time-decay correction (*k* = time-decay coefficient), where *H* is the counts/pixel in the heart and *M* refers to the counts/pixel in the upper mediastinum [21–24]. CSN overactivity was defined as delayed *H*/*M* ratio < 2.2 and WR > 34% following previous studies [16, 23].

### Statistical analyses

Continuous measures are expressed as means ± SDs or medians and interquartile ranges for normally and skewed distributed variables, respectively. Categorical variables are presented as counts and percentages. Continuous variables were compared using the Wilcoxon’s test. Categorical variables were compared between the two groups using the chi-square test or Fisher’s exact test. Pearson’s correlation coefficient was used to evaluate the correlations between the QTc and clinical variables, including delayed *H*/*M* ratio, and WR. In addition, Fisher’s *Z* test was used to evaluate the differences in the strength of the correlations between men and women. Univariate and multivariate logistic regression analyses were conducted to identify the factors associated with CSN overactivity. Variables previously reported to be associated with CSN activity were included in the analysis [25–28]. Potential predictors with *p* < 0.10 on a univariate analysis were included in a multivariate regression analysis. Receiver-operating characteristic (ROC) analysis was performed to evaluate the predictive value of the QTc for identifying CSN overactivity. All statistical analyses were performed using IBM SPSS Statistics version 30 (IBM Corp., Armonk, NY, USA). A two-tailed *p* value of < 0.05 denoted significance.

## Results

### Characteristics of the study participants

Table 1 presents the characteristics of the study participants. The mean age was 84 ± 5 years, and 20 patients (24%) were male. The mean QTc in the entire population was 441 ± 27 ms. Prolonged QTc was found in 14 patients. The mean QTc was 486 ± 15 ms in 14 patients with prolonged QTc and 432 ± 18 ms in those with normal QTc. The prolonged QTc group was more likely to be male (*p* = 0.02) and have intraventricular block (*p* = 0.02) than the normal QTc group. No significant differences in age, heart rate, blood pressure, past medical history, and medication use were observed between the groups. No medication changes were made during the study period. The AVA was similar between the two groups. The prolonged QTc group had higher LVMI (*p* = 0.04) and lower mean pressure gradient (*p* = 0.01) and LVEF (*p* < 0.001) than the normal QTc group.

**Table 1:** Baseline characteristics of patients with prolonged vs normal QTc

	All	Prolonged QTc	Normal QTc	*p* value
Variables	(*n* = 83)	(*n* = 14)	(*n* = 69)	
Age (years)	84 ± 5	84 ± 5	84 ± 5	0.82
Male	20 (24%)	7 (50%)	13 (19%)	0.02
Body mass index (kg/m^2^)	23.2 ± 3.5	23.8 ± 3.8	23.0 ± 3.5	0.48
STS score	7.5 ± 2.3	8.5 ± 3.2	7.4 ± 2.1	0.15
NYHA class III	40 (48%)	10 (71%)	30 (43%)	0.06
Past medical history				
Hypertension	77 (93%)	13 (93%)	64 (93%)	0.99
Diabetes mellitus	9 (11%)	1 (7%)	8 (12%)	0.61
Dyslipidemia	50 (60%)	11 (79%)	39 (57%)	0.11
Coronary artery disease	17 (20%)	2 (14%)	15 (22%)	0.51
Current smoking	2 (2%)	0 (0%)	2 (3%)	0.39
Heart rate	69 ± 10	73 ± 13	68 ± 10	0.09
Systolic blood pressure (mmHg)	130 ± 18	127 ± 23	131 ± 17	0.51
Diastolic blood pressure (mmHg)	68 ± 11	69 ± 13	68 ± 11	0.68
Laboratory data				
Hemoglobin (mg/dL)	11.6 ± 1.4	11.7 ± 1.5	11.6 ± 1.4	0.66
Creatinine (mg/dL)	0.86 ± 0.25	0.97 ± 0.22	0.84 ± 0.24	0.07
NT-proBNP (pg/dL)	1073 (578–2135)	1659 (882–3196)	940 (393–2114)	0.11
Potassium (mmol/L)	4.2 ± 0.4	4.3 ± 0.4	4.2 ± 0.4	0.62
Medications				
ACEIs or ARBs	53 (64%)	10 (71%)	43 (62%)	0.51
Beta blockers	23 (28%)	6 (43%)	17 (25%)	0.18
Diuretics	39 (47%)	8 (57%)	31 (45%)	0.40
Statins	40 (48%)	10 (71%)	30 (43%)	0.06
Electrocardiographic data				
Intraventricular block	11 (13%)	5 (36%)	6 (9%)	0.02
PR interval (ms)	175 ± 27	185 ± 37	173 ± 25	0.12
QRS duration (ms)	98 ± 20	121 ± 23	94 ± 16	< 0.001
QTc (ms)	441 ± 27	486 ± 15	432 ± 18	Not applicable
RV5 + SV1 voltage (mV)	3.80 ± 1.29	3.28 ± 1.28	3.90 ± 1.28	0.10
Transthoracic echocardiographic data				
AVA (cm^2^)	0.63 ± 0.18	0.69 ± 0.16	0.63 ± 0.19	0.30
Mean pressure gradient (mmHg)	57 ± 19	45 ± 17	59 ± 19	0.01
LVMI (g/m^2^)	119 ± 32	132 ± 48	116 ± 27	0.04
LVEF (%)	64 ± 7	57 ± 10	66 ± 5	< 0.001
Severe MR	2 (2%)	0 (0%)	2 (3%)	0.39
TR pressure gradient (mmHg)	29 ± 9	27 ± 7	30 ± 9	0.40
AS subtypes				0.01
High gradient	69 (83%)	10 (71%)	59 (86%)	
Normal flow low gradient	10 (12%)	1 (7%)	9 (13%)	
Classical low flow low gradient	3 (4%)	3 (21%)	0 (0%)	
Paradoxical low flow low gradient	1 (1%)	0 (0%)	1 (1%)	

*ACEI* angiotensin-converting enzyme inhibitor; *ARB* angiotensin II receptor blocker; *AS* aortic stenosis; *AVA* aortic valve area; *LVEF* left ventricular ejection fraction; *LVMI* left ventricular mass index; *MR* mitral regurgitation; *NT-proBNP* N-terminal pro-brain natriuretic peptide; *NYHA* New York Heart Association; *QTc* corrected QT interval; *STS* Society of Thoracic Surgeons; *TR* tricuspid regurgitation

### 123I-MIBG parameters of patients with prolonged vs normal QTc

**Fig. 2:**
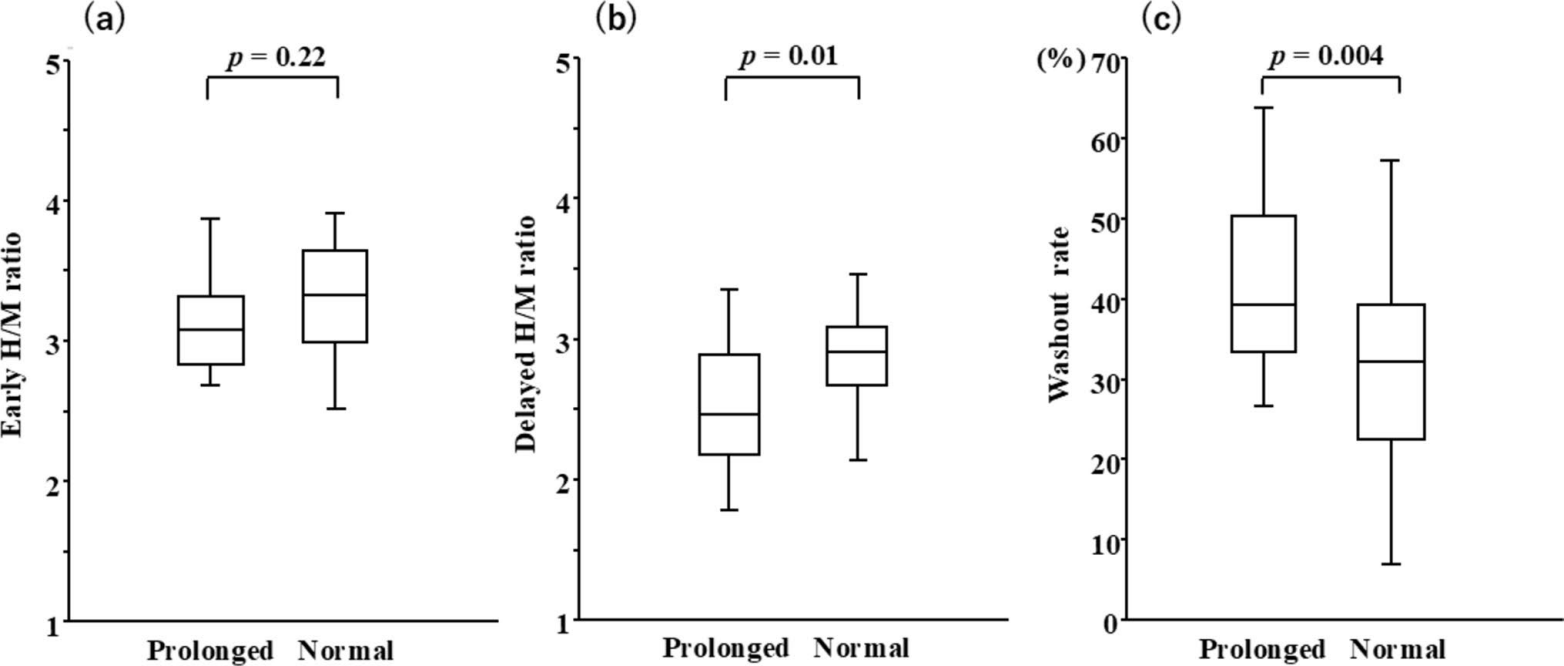
Early *H*/*M* ratio (**a**), delayed *H*/*M* ratio (**b**), and WR (**c**) in patients with prolonged QTc and normal QTc. *H/M* heart-to-mediastinum, *QTc* corrected QT interval. *WR* washout rate

The mean time interval between ECG and 123I-MIBG scintigraphy was 4 ± 2 days. No differences in the time interval between ECG and 123I-MIBG scintigraphy were found between the two groups (4 ± 3 days vs 4 ± 2 days, *p* = 0.51). More patients had CSN overactivity in the prolonged QTc group than in the normal QTc group (*n* = 6 [43%] vs *n* = 9 [13%], *p* = 0.02). No significant difference was observed in the early *H*/*M* ratio between the two groups (3.1 ± 0.3 vs 3.3 ± 0.4, *p* = 0.22). However, the delayed *H*/*M* ratio was lower in the prolonged QTc group than in the normal QTc group (2.5 ± 0.5 vs 2.9 ± 0.4, *p* = 0.01), whereas the WR was higher in the prolonged QTc group than in the normal QTc group (40 ± 11% vs 32 ± 11%, *p* = 0.004) (Table 2, Fig. 2).

**Table 2:** 123I-MIBG parameters of patients with prolonged vs normal QTc

	All	Prolonged QTc	Normal QTc	*p* Value
Variables	(*n* = 83)	(*n* = 14)	(*n* = 69)	
Early *H/M* ratio	3.2 ± 0.4	3.1 ± 0.3	3.3 ± 0.4	0.22
Delayed *H/M* ratio	2.8 ± 0.5	2.5 ± 0.5	2.9 ± 0.4	0.01
WR (%)	33 ± 12	40 ± 11	32 ± 11	0.004

*H/M* heart-to-mediastinum, *QTc* corrected QT interval, *WR* washout rate

### Relationship between the QTc and 123I-MIBG parameters

**Fig. 3:**
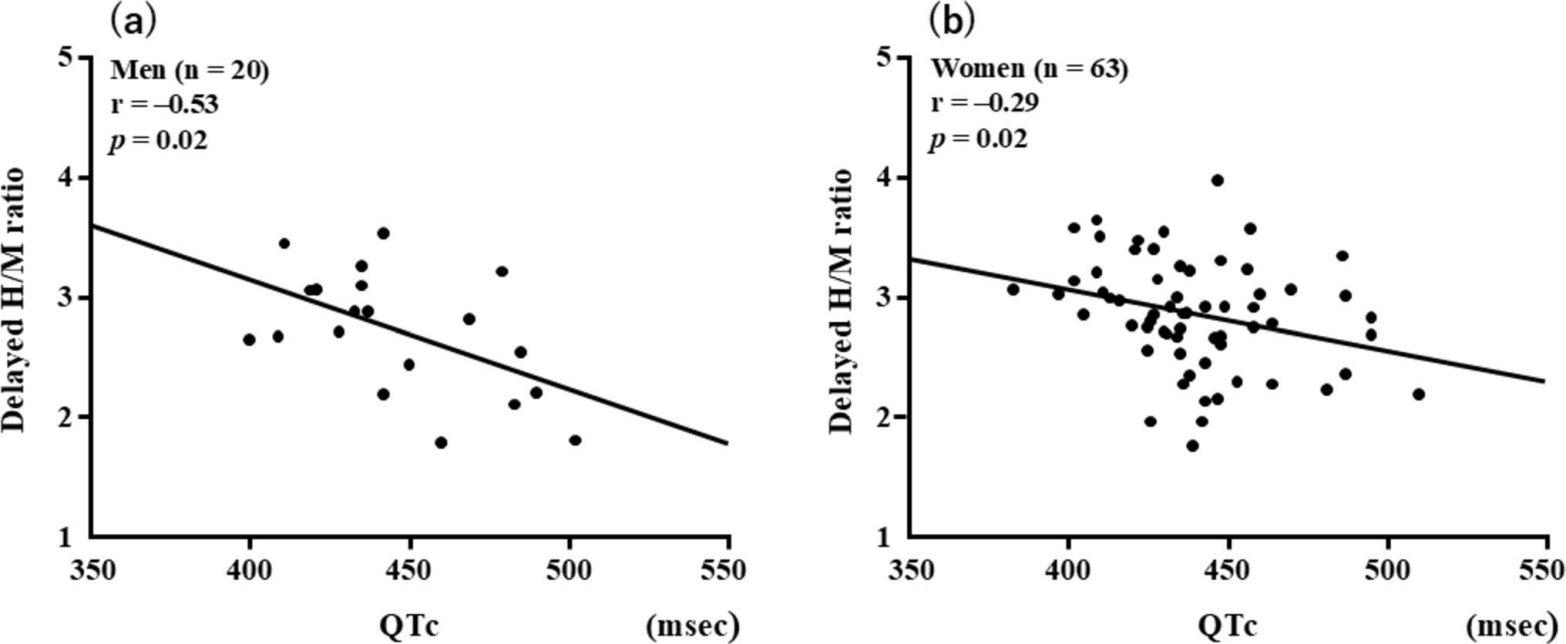
Correlations between QTc and delayed *H*/*M* ratio in men (**a**) and women (**b**). *H/M* heart-to-mediastinum, *QT* corrected QT interval

**Fig. 4:**
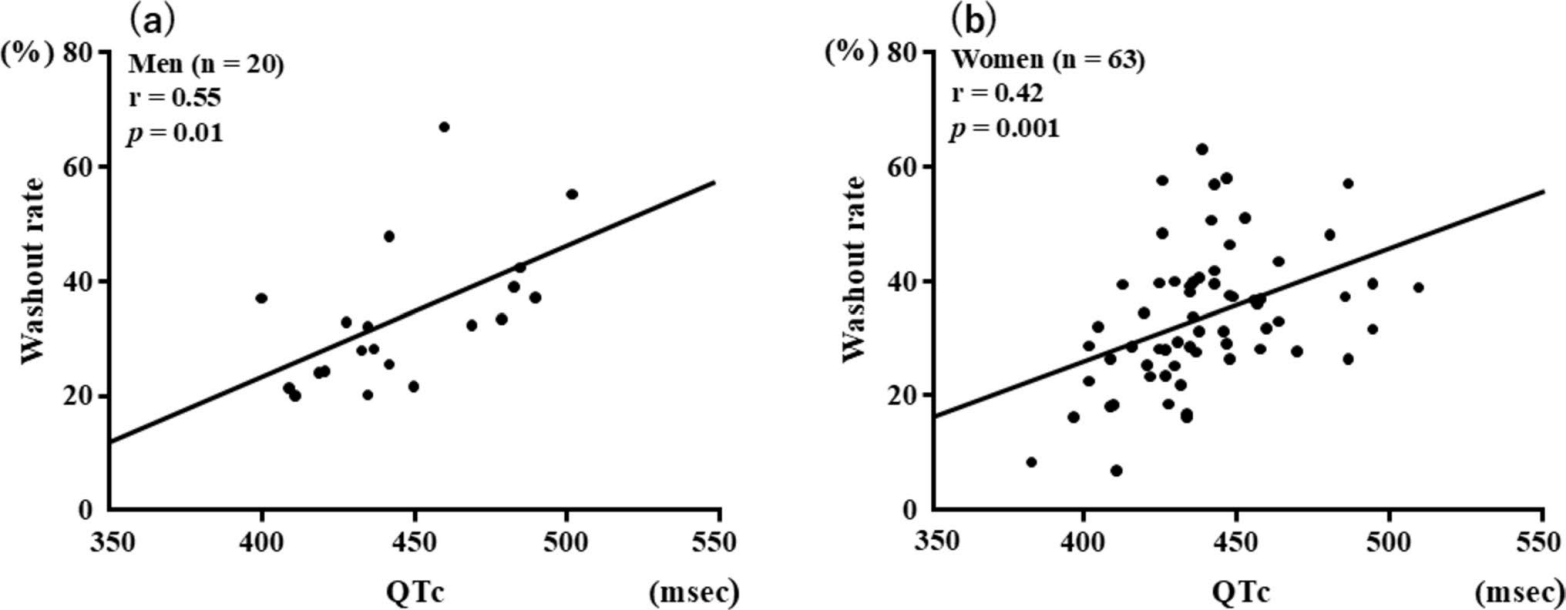
Correlations between QTc and WR in men (**a**) and women (**b**). *QTc* corrected QT interval, *WR* washout rate

**Fig. 5:**
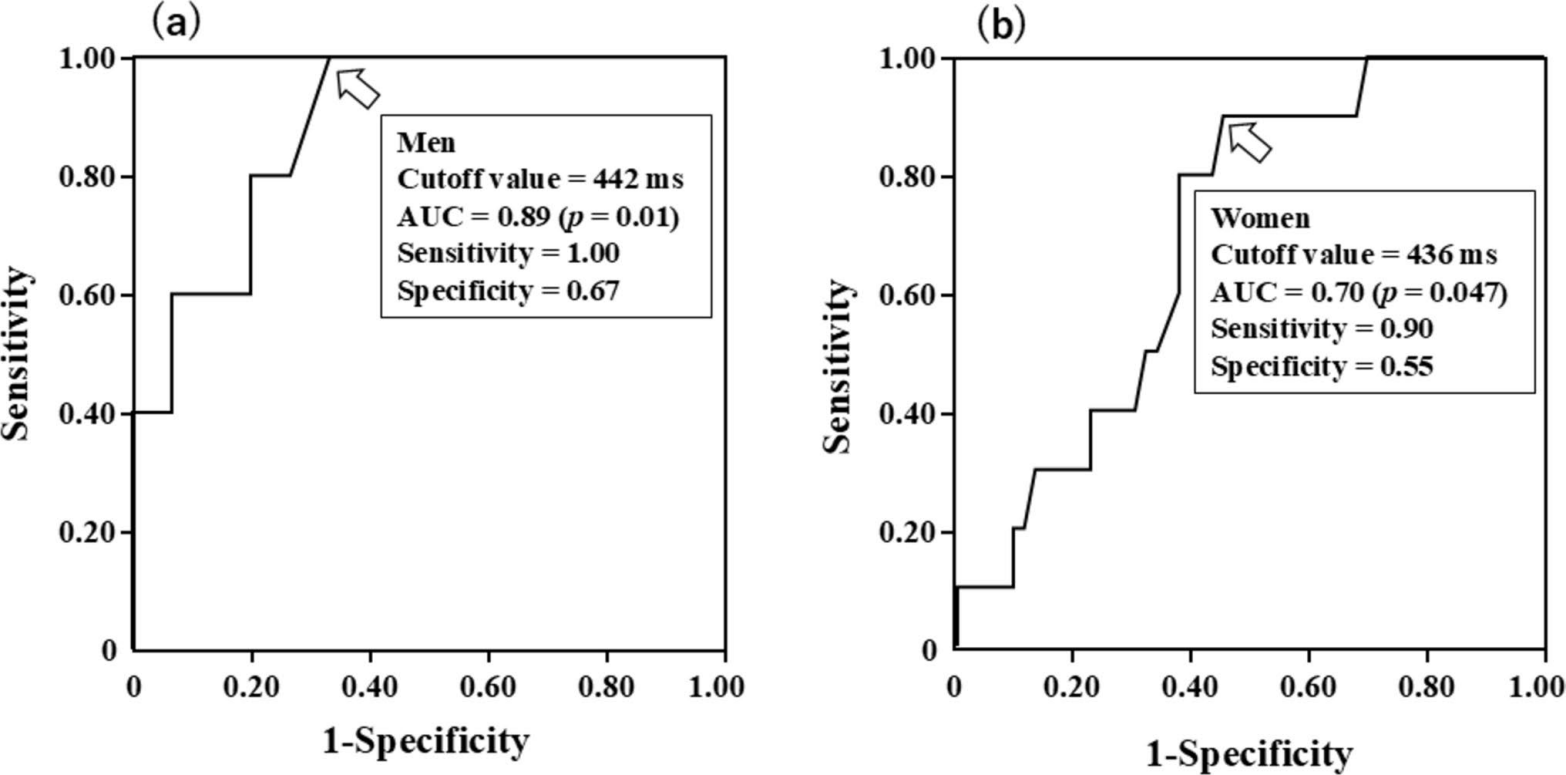
ROC analysis to describe the relationship between QTc and the presence of CSN overactivity in men (**a**) and women (**b**). *CSN* cardiac sympathetic nervous, *QTc* corrected QT interval, *ROC* receiver operating characteristic

QTc was inversely correlated with the delayed *H*/*M* ratio in both men (*r* = − 0.53 [95% CI − 0.77 to − 0.88], *p* = 0.02) and women (*r* = − 0.29 [95% CI − 0.50 to − 0.04], *p* = 0.02) (Fig. 3). QTc was positively correlated with WR in both men (*r* = 0.55 [95% CI 0.09–0.78], *p* = 0.01) and women (*r* = 0.42 [95% CI 0.19–0.60], *p* = 0.001). However, no differences in the strength of the correlations were found between men and women (delayed *H*/*M* ratio, *p* = 0.39; WR, *p* = 0.83) (Fig. 4). Multivariate logistic regression revealed that age and prolonged QTc were significantly associated with CSN overactivity (Table 3). ROC analysis revealed cutoffs for QTc of 442 ms in men (area under the curve [AUC], 0.89, 95% CI 0.73–1.04, *p* = 0.01) and 436 ms in women (AUC, 0.70, 95% CI 0.55–0.85, *p* = 0.047) to predict CSN overactivity (Fig. 5).

**Table 3:** Univariate and multivariate logistic regression analyses to identify factors associated with the CSN overactivity

	Univariate		Multivariate (Model 1)		Multivariate (Model 2)	
Variables	OR (95% CI)	*p* Value	OR (95% CI)	*p* Value	OR (95% CI)	*p* Value
Age (years)	1.16 (1.01–1.36)	0.03	1.19 (1.02–1.44)	0.02	1.15 (1.01–1.37)	0.048
Male gender	1.77 (0.49–5.82)	0.37				
NYHA class III	2.53 (0.80–8.87)	0.11				
Coronary artery disease	1.54 (0.38–5.36)	0.52				
ACEIs or ARBs	0.82 (0.26–2.70)	0.73				
Beta blockers	0.60 (0.13–2.14)	0.45				
Diuretics	1.36 (0.44–4.30)	0.59				
AVA (cm^2^)	1.97 (0.08–45.4)	0.66				
LVMI (g/m^2^)	1.02 (1.00–1.04)	0.01	1.01 (0.99–1.04)	0.16	1.01 (0.99–1.04)	0.17
LVEF (%)	0.93 (0.86–1.00)	0.06	1.06 (0.95–1.21)	0.31	1.01 (0.92–1.13)	0.82
QTc (per 10 ms increase)	1.38 (1.13–1.75)	0.001	1.53 (1.16–2.16)	0.002		
Prolonged QTc	5.00 (1.38–18.1)	0.02			5.14 (1.13–28.3)	0.046

*ACEI* angiotensin-converting enzyme inhibitor; *ARB* angiotensin II receptor blocker; *AVA* aortic valve area; *LVEF* left ventricular ejection fraction; *LVMI* left ventricular mass index; *NYHA* New York Heart Association; *QTc* corrected QT interval

## Discussion

The major findings of this study were as follows: (1) CSN overactivity was more prevalent in patients with prolonged QTc than in those with normal QTc, (2) QTc was inversely correlated with the delayed *H*/*M* ratio and positively correlated with the WR, and (3) Prolonged QTc was an independent predictor of CSN overactivity in patients with severe AS. These results indicate that serial monitoring with ECG may noninvasively predict the presence of CSN overactivity and provide useful information for the management of patients with severe AS.

Prolonged QTc has been recognized as a marker of advanced disease, associated with an adverse hemodynamic profile and increased long-term mortality following aortic valve replacement [13, 14]. However, in this study, these correlations were relatively weak, suggesting that QTc prolongation is not simply a surrogate for more severe cardiac dysfunction. Sun et al. [29] reported that prolonged QTc and CSN overactivity were associated with LV remodeling in a chronic pressure overload rat model of heart failure, suggesting that CSN dysfunction may underlie the association between QTc and mortality. In the present study, 123I-MIBG scintigraphy was employed to evaluate the association between QTc and CSN activity in patients with severe AS; thereby, indicating a link between QTc prolongation and CSN overactivity.

In this study, CSN overactivity was defined as delayed *H*/*M* ratio < 2.2 and WR > 34%, in accordance with a previous study [23] reporting higher norepinephrine levels in the reduced delayed *H*/*M* ratio group (< 2.2) when compared with the preserved delayed *H*/*M* ratio group. Prolonged QTc was significantly associated with CSN overactivity. However, prolonged QTc was no longer a statistically significant predictor of CSN overactivity when using the cutoff of 2.0 for delayed *H*/*M* ratio (Table S1). This discrepancy might have been due to the difference in number of patients diagnosed with CSN overactivity between analyses. Further studies with a larger sample size are necessary to establish the lower limit of normal delayed *H*/*M* ratio in patients with AS.

CSN activity can rapidly respond to hemodynamic changes and is susceptible to other clinical factors. In this context, the high WR with a normal delayed *H*/*M* ratio may not reflect persistent CSN overactivity. Given that WR reflects dynamic sympathetic tone, it was used in conjunction with delayed *H*/*M* ratio to define CSN overactivity in this study. Sobajima et al. [23] reported that the mean WR before TAVR was 34% when using low- to medium-energy general-purpose collimators. Similarly, Nakajima et al. [16] reported that WR normally ranges from 0% to 34%. Therefore, a WR of 34% was the cutoff value in the present study.

Several potential mechanisms may explain the present findings. Although knowledge of the role of myocardial fibrosis in AS is evolving [30–32], the known associations between QTc prolongation and myocardial fibrosis may be an underlying mechanism. Riza Demir et al. [33] demonstrated a correlation between QTc, LVMI and myocardial fibrosis in patients with hypertrophic cardiomyopathy. Additionally, autonomic innervation density may be adversely affected by myocardial fibrosis. Da Silva et al*.* [34] indicated that CSN overactivity is associated with extracellular volume expansion, a surrogate marker of myocardial fibrosis, in patients with severe AS. Consistent with these findings, the present study revealed that patients with prolonged QTc had higher LVMI and more frequent CSN overactivity. These results suggest that myocardial fibrosis, which is associated with prolonged QTc, results in LV remodeling and dysfunction and, consequently, CSN overactivity.

The association between QTc prolongation and reduced cardiovagal baroreflex sensitivity is another possible mechanism. Tschumper et al*.* [13] reported that patients with prolonged QTc and severe AS often exhibit low mean aortic pressure. The baroreceptors located in the aortic arch can detect the stretching of the aortic wall during an increase in aortic pressure, which helps to control blood pressure by suppressing CSN activity. However, in patients with prolonged QTc, the baroreceptors may not be sufficiently loaded because of low aortic pressure. Consequently, the reduction in CSN activity mediated by the baroreflex is insufficient, leading to CSN overactivity. In contrast, many investigators have reported associations between QTc and the intimal–medial thickness of the carotid artery, another important barosensory artery [35–37]. In addition, Nakaya et al. [38] reported severe AS was an independent predictor for carotid atherosclerosis. Previously, we reported that sympathetic and cardiovagal baroreflex sensitivities were inversely correlated with carotid artery stiffness [39]. Therefore, patients with AS exhibiting prolonged QTc may also have high carotid artery stiffness, which leads to a reduction in cardiovagal baroreflex sensitivity and, consequently, CSN overactivity.

This study has several limitations that should be acknowledged. First, the effects of 123I-MIBG parameters on clinical outcomes in patients with AS remain unclear. Further studies with a larger sample size and across a wider range of AS severity levels are necessary to establish the prognostic implications of 123I-MIBG parameters in patients with AS. Second, coronary microvascular function, such as fractional flow reserve and index of microvascular resistance, were not evaluated. Recently, Schipaanboord et al*.* [40] reported an association between prolonged QTc and angina and nonobstructed coronary arteries, which is mainly caused by microvascular dysfunction. Third, the possibility of cardiac amyloidosis in this study population could not be excluded. Fourth, 123I-MIBG scintigraphy was indicated for heart failure evaluation, thus, there may have been a selection bias. Fifth, the WR threshold used in the analysis is not standardized and can vary depending on the corrections applied during the analysis [16, 41]. Finally, the small sample size is a major limitation of this study.

## Conclusions

Prolonged QTc is associated with CSN overactivity in patients with severe AS, as assessed using 123I-MIBG scintigraphy.

## Supplementary Information

Below is the link to the electronic supplementary material.

Supplementary file1 (DOCX 20 KB)

Supplementary file2 (DOCX 19 KB)

Supplementary file3 (DOCX 20 KB)

## Data Availability

The data that support the findings of this study are available from the corresponding author upon reasonable request.

## References

[1] Zile MR, Gaasch WH (2003) Heart failure in aortic stenosis—improving diagnosis and treatment. N Engl J Med 348:1735–1736 12724478 10.1056/NEJMp030035

[2] Maganti K, Rigolin VH, Sarano ME, Bonow RO (2010) Valvular heart disease: diagnosis and management. Mayo Clin Proc 85:483–500 20435842 10.4065/mcp.2009.0706 PMC2861980

[3] Dumonteil N, Vaccaro A, Despas F, Labrunee M, Marcheix B, Lambert E, Esler M, Carrie D, Senard JM, Galinier M, Pathak A (2013) Transcatheter aortic valve implantation reduces sympathetic activity and normalizes arterial spontaneous baroreflex in patients with aortic stenosis. JACC Cardiovasc Interv 6:1195–1202 24139928 10.1016/j.jcin.2013.06.012

[4] Jacobson AF, Senior R, Cerqueira MD, Wong ND, Thomas GS, Lopez VA, Agostini D, Weiland F, Chandna H, Narula J (2010) Myocardial iodine-123 meta-iodobenzylguanidine imaging and cardiac events in heart failure. Results of the prospective ADMIRE-HF (AdreView myocardial imaging for risk evaluation in heart failure) study. J Am Coll Cardiol 55:2212–2221 20188504 10.1016/j.jacc.2010.01.014

[5] Nakata T, Nakajima K, Yamashina S, Yamada T, Momose M, Kasama S, Matsui T, Matsuo S, Travin MI, Jacobson AF (2013) A pooled analysis of multicenter cohort studies of (123)I-mIBG imaging of sympathetic innervation for assessment of long-term prognosis in heart failure. JACC Cardiovasc Imaging 6:772–784 23845574 10.1016/j.jcmg.2013.02.007

[6] Tamaki S, Yamada T, Okuyama Y, Morita T, Sanada S, Tsukamoto Y, Masuda M, Okuda K, Iwasaki Y, Yasui T, Hori M, Fukunami M (2009) Cardiac iodine-123 metaiodobenzylguanidine imaging predicts sudden cardiac death independently of left ventricular ejection fraction in patients with chronic heart failure and left ventricular systolic dysfunction: results from a comparative study with signal-averaged electrocardiogram, heart rate variability, and QT dispersion. J Am Coll Cardiol 53:426–435 19179201 10.1016/j.jacc.2008.10.025

[7] Egi R, Fukushima K, Matsusaka Y, Yamane T, Seto A, Matsunari I, Nakajima Y, Nakano S, Kuji I (2024) Cardiac sympathetic nerve function in patients with severe aortic stenosis prior and after transcatheter aortic valve implantation: evaluation by 5-year risk model. Ann Nucl Cardiol 10:6–15 39635331 10.17996/anc.23-00008 PMC11612390

[8] Kadoya Y, Zen K, Tamaki N, Yashige M, Takamatsu K, Ito N, Kuwabara K, Yamano M, Yamano T, Nakamura T, Yaku H, Matoba S (2021) Prognostic value of cardiac (123) I-metaiodobenzylguanidine imaging for predicting cardiac events after transcatheter aortic valve replacement. ESC Heart Fail 8:1106–1116 33400391 10.1002/ehf2.13123 PMC8006649

[9] Solti F, Szatmáry L, Vecsey T, Szabolcs Z (1989) The effect of sympathetic and parasympathetic activity on QT duration. Clinical study in patients with normal and prolonged QT time. Cor Vasa 31:9–15 2721207

[10] Lai S, Perrotta AM, Bagordo D, Mazzaferro S, Menè P, Gigante A, Tinti F, Galani A, Cianci R (2021) Screening of QTc interval and global autonomic activity in autosomal dominant polycystic kidney disease and atherosclerotic renal artery stenosis hypertensive patients. Eur Rev Med Pharmacol Sci 25:6333–6338 34730214 10.26355/eurrev_202110_27005

[11] Marfella R, Gualdiero P, Siniscalchi M, Carusone C, Verza M, Marzano S, Esposito K, Giugliano D (2003) Morning blood pressure peak, QT intervals, and sympathetic activity in hypertensive patients. Hypertension 41:237–243 12574088 10.1161/01.hyp.0000050651.96345.0e

[12] Bienias P, Ciurzyński M, Kisiel B, Chrzanowska A, Ciesielska K, Siwicka M, Kalińska-Bienias A, Saracyn M, Lisicka M, Radochońska J, Pruszczyk P (2019) Comparison of non-invasive assessment of arrhythmias, conduction disturbances and cardiac autonomic tone in systemic sclerosis and systemic lupus erythematosus. Rheumatol Int 39:301–310 30421103 10.1007/s00296-018-4207-x

[13] Tschumper M, Weber L, Rickli H, Seidl S, Brenner R, Buser M, Ehl NF, Jäger-Rhomberg F, Ammann P, Maeder MT (2021) Corrected QT Interval in severe aortic stenosis: clinical and hemodynamic correlates and prognostic impact. Am J Med 134:267–277 32621909 10.1016/j.amjmed.2020.05.035

[14] Dahou A, Toubal O, Clavel MA, Beaudoin J, Magne J, Mathieu P, Philippon F, Dumesnil JG, Puri R, Ribeiro HB, Larose É, Rodés-Cabau J, Pibarot P (2016) Relationship between QT interval and outcome in low-flow low-gradient aortic stenosis with low left ventricular ejection fraction. J Am Heart Assoc 5:e003980 27792655 10.1161/JAHA.116.003980 PMC5121501

[15] Baumgartner H, Hung J, Bermejo J, Chambers JB, Edvardsen T, Goldstein S, Lancellotti P, LeFevre M, Miller F Jr, Otto CM (2017) Recommendations on the echocardiographic assessment of aortic valve stenosis: a focused update from the European Association of Cardiovascular Imaging and the American Society of Echocardiography. J Am Soc Echocardiogr 30:372–392 28385280 10.1016/j.echo.2017.02.009

[16] Nakajima K, Matsumoto N, Kasai T, Matsuo S, Kiso K, Okuda K (2016) Normal values and standardization of parameters in nuclear cardiology: Japanese Society of Nuclear Medicine working group database. Ann Nucl Med 30:188–199 26897008 10.1007/s12149-016-1065-z PMC4819542

[17] Jacobson AF, Travin MI (2015) Impact of medications on mIBG uptake, with specific attention to the heart: comprehensive review of the literature. J Nucl Cardiol 22:980–993 25975946 10.1007/s12350-015-0170-z

[18] Nitta K, Kurisu S, Nakamoto Y, Sumimoto Y, Senoo A, Ikenaga H, Tatsugami F, Ishibashi K, Kitagawa T, Fukuda Y, Yamamoto H, Awai K, Kihara Y (2019) Coronary artery calcium is associated with left ventricular diastolic function independent of myocardial ischemia. Int Heart J 60:554–559 31105144 10.1536/ihj.18-355

[19] Lang RM, Badano LP, Mor-Avi V, Afilalo J, Armstrong A, Ernande L, Flachskampf FA, Foster E, Goldstein SA, Kuznetsova T, Lancellotti P, Muraru D, Picard MH, Rietzschel ER, Rudski L, Spencer KT, Tsang W, Voigt JU (2015) Recommendations for cardiac chamber quantification by echocardiography in adults: an update from the American Society of Echocardiography and the European Association of Cardiovascular Imaging. Eur Heart J Cardiovasc Imaging 16:233–270 25712077 10.1093/ehjci/jev014

[20] Takahari K, Utsunomiya H, Itakura K, Yamamoto H, Nakano Y (2022) Impact of the distribution of epicardial and visceral adipose tissue on left ventricular diastolic function. Heart Vessels 37:250–261 34228157 10.1007/s00380-021-01904-0

[21] Nitta K, Fukuda Y, Susawa H, Ikenaga H, Utsunomiya H, Ishibashi K, Kurisu S, Takahashi S, Nakano Y, Awai K, Sueda T, Kihara Y (2020) Impact of prosthesis-patient mismatch after transcatheter aortic valve replacement on changes in cardiac sympathetic nervous function. Int Heart J 61:1188–1195 33191358 10.1536/ihj.20-381

[22] Higashihara T, Fukuda Y, Nakano T, Takeda A, Morita Y, Ono M, Watanabe N, Sada Y, Ikenaga H, Utsunomiya H, Takahashi S, Nakano Y (2023) Left-atrial volume reduction reflects improvement of cardiac sympathetic nervous function in patients with severe aortic stenosis after transcatheter aortic valve replacement. Heart Vessels 38:1083–1091 36928668 10.1007/s00380-023-02257-6

[23] Sobajima M, Ueno H, Onoda H, Kuwahara H, Tanaka S, Ushijima R, Fukuda N, Yokoyama S, Nagura S, Doi T, Yamashita A, Fukahara K, Ito H, Kinugawa K (2018) Transcatheter aortic valve implantation improves cardiac sympathetic nerve activity on (123)I-metaiodobenzylguanidine myocardial scintigraphy in severe aortic valve stenosis. Circ J 82:579–585 28966286 10.1253/circj.CJ-17-0817

[24] Kadoya Y, Zen K, Tamaki N, Ito N, Kuwabara K, Yamano M, Yamano T, Nakamura T, Matsushima S, Oka K, Numata S, Yaku H, Matoba S (2020) Early effects of transcatheter aortic valve replacement on cardiac sympathetic nervous function assessed by (123)I-metaiodobenzylguanidine scintigraphy in patients with severe aortic valve stenosis. Eur J Nucl Med Mol Imaging 47:1657–1667 31502014 10.1007/s00259-019-04523-0

[25] Nitta K, Fukuda Y, Takahari K, Takeda A, Higashihara T, Morita Y, Watanabe N, Ikenaga H, Utsunomiya H, Ishibashi K, Kurisu S, Takahashi S, Awai K, Nakano Y (2022) Factors influencing cardiac sympathetic nervous function in patients with severe aortic stenosis: assessment by (123)I-metaiodobenzylguanidine myocardial scintigraphy. Heart Lung Circ 31:671–677 34794871 10.1016/j.hlc.2021.09.022

[26] Kasama S, Toyama T, Kumakura H, Takayama Y, Ichikawa S, Suzuki T, Kurabayashi M (2005) Effects of candesartan on cardiac sympathetic nerve activity in patients with congestive heart failure and preserved left ventricular ejection fraction. J Am Coll Cardiol 45:661–667 15734608 10.1016/j.jacc.2004.11.038

[27] de Peuter OR, Verberne HJ, Kok WE, van den Bogaard B, Schaap MC, Nieuwland R, Meijers JC, Somsen GA, Bakx A, Kamphuisen PW (2013) Differential effects of nonselective versus selective β-blockers on cardiac sympathetic activity and hemostasis in patients with heart failure. J Nucl Med 54:1733–1739 23970363 10.2967/jnumed.113.120477

[28] Francis GS, Siegel RM, Goldsmith SR, Olivari MT, Levine TB, Cohn JN (1985) Acute vasoconstrictor response to intravenous furosemide in patients with chronic congestive heart failure. Activation of the neurohumoral axis. Ann Intern Med 103:1–6 2860833 10.7326/0003-4819-103-1-1

[29] Sun F, Yuan L, Wang Z, Cui X, Lv N, Zhang T, Zhang Y, Cai J (2024) Cardiac sympathetic overdrive, M2 macrophage activation and fibroblast heterogeneity are associated with cardiac remodeling in a chronic pressure overload rat model of HFpEF. Front Pharmacol 15:1364758 38860171 10.3389/fphar.2024.1364758 PMC11163040

[30] Barone-Rochette G, Piérard S, Meester De, de Ravenstein C, Seldrum S, Melchior J, Maes F, Pouleur AC, Vancraeynest D, Pasquet A, Vanoverschelde JL, Gerber BL (2014) Prognostic significance of LGE by CMR in aortic stenosis patients undergoing valve replacement. J Am Coll Cardiol 64:144–154 25011718 10.1016/j.jacc.2014.02.612

[31] Evertz R, Hub S, Beuthner BE, Backhaus SJ, Lange T, Schulz A, Toischer K, Seidler T, von Haehling S, Puls M, Kowallick JT, Zeisberg EM, Hasenfuß G, Schuster A (2023) Aortic valve calcification and myocardial fibrosis determine outcome following transcatheter aortic valve replacement. ESC Heart Fail 10:2307–2318 37060191 10.1002/ehf2.14307 PMC10375183

[32] Barone-Rochette G, Piérard S, Seldrum S, de Meester de Ravenstein C, Melchior J, Maes F, Pouleur AC, Vancraeynest D, Pasquet A, Vanoverschelde JL, Gerber BL (2013) Aortic valve area, stroke volume, left ventricular hypertrophy, remodeling, and fibrosis in aortic stenosis assessed by cardiac magnetic resonance imaging: comparison between high and low gradient and normal and low flow aortic stenosis. Circ Cardiovasc Imaging 6:1009–1017 24100045 10.1161/CIRCIMAGING.113.000515

[33] Riza Demir A, Celik Ö, Sevinç S, Uygur B, Kahraman S, Yilmaz E, Cemek M, Onal Y, Erturk M (2019) The relationship between myocardial fibrosis detected by cardiac magnetic resonance and Tp-e interval, 5-year sudden cardiac death risk score in hypertrophic cardiomyopathy patients. Ann Noninvasive Electrocardiol 24:e12672 31152489 10.1111/anec.12672 PMC6931578

[34] da Silva LM, Coy-Canguçu A, Paim LR, Bau AA, Nicolela Geraldo Martins C, Pinheiro S, Citeli Ribeiro V, Magalhães Rocha WE, Mattos-Souza JR, Schreiber R, Antunes-Correa L, Sposito A, Nadruz W Jr, Ramos CD, Neilan T, Jerosch-Herold M, Coelho-Filho OR (2024) Impaired cardiac sympathetic activity is associated with myocardial remodeling and established biomarkers of heart failure. J Am Heart Assoc 13:e035264 38958130 10.1161/JAHA.124.035264 PMC11292752

[35] Strohmer B, Pichler M, Iglseder B, Paulweber B (2005) Relationship of QT interval duration with carotid intima media thickness in a clinically healthy population undergoing cardiovascular risk screening. J Intern Med 257:238–246 15715680 10.1111/j.1365-2796.2004.01436.x

[36] Deng C, Niu J, Xuan L, Zhu W, Dai H, Zhao Z, Li M, Lu J, Xu Y, Chen Y, Wang W, Ning G, Bi Y, Xu M, Wang T (2020) Association of QTc interval with risk of cardiovascular diseases and related vascular traits: a prospective and longitudinal analysis. Glob Heart 15:13 32489786 10.5334/gh.533 PMC7218767

[37] Takebayashi K, Aso Y, Matsutomo R, Wakabayashi S, Inukai T (2004) Association between the corrected QT intervals and combined intimal-medial thickness of the carotid artery in patients with type 2 diabetes. Metabolism 53:1152–1157 15334377 10.1016/j.metabol.2004.03.018

[38] Nakaya H, Yokoyama N, Watanabe Y, Kataoka A, Konno K, Kozuma K (2020) Prevalence and predictors of atherosclerotic peripheral arterial obstructive disease in severe heart valve diseases. Int Heart J 61:727–733 32684599 10.1536/ihj.20-009

[39] Okada Y, Galbreath MM, Shibata S, Jarvis SS, VanGundy TB, Meier RL, Vongpatanasin W, Levine BD, Fu Q (2012) Relationship between sympathetic baroreflex sensitivity and arterial stiffness in elderly men and women. Hypertension 59:98–104 22106403 10.1161/HYPERTENSIONAHA.111.176560 PMC3241909

[40] Schipaanboord DJM, Jansen TPJ, Crooijmans C, Onland-Moret NC, Elias-Smale SE, Dimitriu-Leen AC, van der Harst P, van de Hoef TP, van Es R, Damman P, den Ruijter HM (2024) ANOCA patients with and without coronary vasomotor dysfunction present with limited electrocardiographic remodeling. Int J Cardiol Heart Vasc 50:101347 38322017 10.1016/j.ijcha.2024.101347 PMC10844962

[41] Verschure DO, Nakajima K, Verberne HJ (2022) Cardiac (123)I-mIBG imaging in heart failure. Pharmaceuticals (Basel) 15:656 35745574 10.3390/ph15060656 PMC9230638

