# Supplementary material for: Prolonged corrected QT interval is associated with cardiac sympathetic nervous function overactivity in patients with severe aortic stenosis: assessment by 123I-metaiodobenzylguanidine myocardial scintigraphy
Source: Heart Vessels. 2025 May 11;40(11):1048–57. doi: 10.1007/s00380-025-02550-6 (PMC12532630; doi:10.1007/s00380-025-02550-6)
Supplement: Supplementary file 1 — Supplementary file1 (DOCX 20 KB) [file 380_2025_2550_MOESM1_ESM.docx]

| Table S1 Univariate and multivariate logistic regression analyses to identify factors associated with the CSN overactivity (delayed H/M ratio <2.0) | | | | |
| --- | --- | --- | --- | --- |
|  | Univariate | | Multivariate | |
| Variables | OR (95% CI) | *p* value | OR (95% CI) | *p* value |
| Age (years) | 1.27 (1.00-1.75) | 0.048 | 1.22 (0.97-1.64) | 0.09 |
| Male gender | 2.22 (0.28-14.4) | 0.42 |  |  |
| NYHA class III | 5.99 (0.91-117.76) | 0.06 | 5.55 (0.83-109.59) | 0.09 |
| Coronary artery disease | 0.97 (0.05-7.13) | 0.98 |  |  |
| ACEIs or ARBs | 0.35 (0.04-2.25) | 0.26 |  |  |
| Beta blockers | 0.59 (0.03-4.32) | 0.60 |  |  |
| Diuretics | 0.74 (0.09-4.69) | 0.75 |  |  |
| AVA (cm^2^) | 31.1 (0.19-11414.16) | 0.19 |  |  |
| LVMI (g/m^2^) | 1.02 (0.99-1.04) | 1.00 |  |  |
| LVEF (%) | 0.93 (0.84-1.05) | 0.77 |  |  |
| QTc (per 10 ms increase) | 1.16 (0.83-1.59) | 0.99 |  |  |
| Prolonged QTc | 3.67 (0.45-24.5) | 0.20 |  |  |
| *ACEI* angiotensin-converting enzyme inhibitor, *ARB* angiotensin II receptor blocker, *AVA* aortic valve area, *LVEF* left ventricular ejection fraction, *LVMI* left ventricular mass index, *NYHA* New York Heart Association, *QTc* corrected QT interval | | | | |
