# Supplementary material for: Prolonged corrected QT interval is associated with cardiac sympathetic nervous function overactivity in patients with severe aortic stenosis: assessment by 123I-metaiodobenzylguanidine myocardial scintigraphy
Source: Heart Vessels. 2025 May 11;40(11):1048–57. doi: 10.1007/s00380-025-02550-6 (PMC12532630; doi:10.1007/s00380-025-02550-6)
Supplement: Supplementary file 2 — Supplementary file2 (DOCX 19 KB) [file 380_2025_2550_MOESM2_ESM.docx]

| Table S2 Univariate and multivariate logistic regression analyses to identify factors associated with the CSN overactivity | | | | |
| --- | --- | --- | --- | --- |
|  | Univariate | | Multivariate | |
| Variables | OR (95% CI) | *p* value | OR (95% CI) | *p* value |
| Age (years) | 1.16 (1.01-1.36) | 0.03 | 1.20 (1.02-1.44) | 0.04 |
| Male gender | 1.77 (0.49-5.82) | 0.37 |  |  |
| NYHA class III | 2.53 (0.80-8.87) | 0.11 |  |  |
| Coronary artery disease | 1.54 (0.38-5.36) | 0.52 |  |  |
| ACEIs or ARBs | 0.82 (0.26-2.70) | 0.73 |  |  |
| Beta blockers | 0.60 (0.13-2.14) | 0.45 |  |  |
| Diuretics | 1.36 (0.44-4.30) | 0.59 |  |  |
| AVA (cm^2^) | 1.97 (0.08-45.4) | 0.66 |  |  |
| LVMI (g/m^2^) | 1.02 (1.00-1.04) | 0.01 | 1.02 (0.99-1.04) | 0.17 |
| LVEF (%) | 0.93 (0.86-1.00) | 0.06 | 1.05 (0.94-1.18) | 0.38 |
| QRS duration (ms) | 1.03 (1.00-1.06) | 0.03 | 1.01 (0.97-1.04) | 0.70 |
| QTc (ms) | 1.03 (1.01-1.06) | 0.002 | 1.04 (1.01-1.08) | 0.02 |
| *ACEI* angiotensin-converting enzyme inhibitor, *ARB* angiotensin II receptor blocker, *AVA* aortic valve area, *LVEF* left ventricular ejection fraction, *LVMI* left ventricular mass index, *NYHA* New York Heart Association, *QTc* corrected QT interval | | | | |
