# Supplementary material for: Prolonged corrected QT interval is associated with cardiac sympathetic nervous function overactivity in patients with severe aortic stenosis: assessment by 123I-metaiodobenzylguanidine myocardial scintigraphy
Source: Heart Vessels. 2025 May 11;40(11):1048–57. doi: 10.1007/s00380-025-02550-6 (PMC12532630; doi:10.1007/s00380-025-02550-6)
Supplement: Supplementary file 3 — Supplementary file3 (DOCX 20 KB) [file 380_2025_2550_MOESM3_ESM.docx]

| Table S3 Univariate logistic regression analyses to identify factors associated with the CSN overactivity | | |
| --- | --- | --- |
|  | Univariate | |
| Variables | OR (95% CI) | *p* value |
| Age (years) | 1.16 (1.01-1.36) | 0.03 |
| Male | 1.77 (0.49-5.82) | 0.37 |
| Body mass index (kg/m^2^) | 0.97 (0.83-1.47) | 0.63 |
| STS score | 1.07 (0.81-1.38) | 0.61 |
| NYHA class III | 2.53 (0.80-8.87) | 0.11 |
| Past medical history |  |  |
| Hypertension | 0.41 (0.07-3.14) | 0.35 |
| Diabetes mellitus | 0.54 (0.03-3.28) | 0.54 |
| Dyslipidemia | 0.71 (0.23-2.24) | 0.55 |
| Coronary artery disease | 1.54 (0.38-5.36) | 0.52 |
| Current Smoking | 1.10e-6 ( -7.47) | 0.37 |
| Heart rate | 0.99 (0.94-1.00) | 0.98 |
| Systolic blood pressure (mmHg) | 1.00 (0.96-1.03) | 0.78 |
| Diastolic blood pressure (mmHg) | 0.99 (0.94-1.04) | 0.60 |
| Laboratory data |  |  |
| Hemoglobin (mg/dL) | 1.20 (0.80-1.88) | 0.39 |
| Creatinine (mg/dL) | 1.32 (0.13-12.6) | 0.81 |
| Log-NT-proBNP | 1.95 (1.08-3.87) | 0.03 |
| Potassium (mmol/L) | 0.47 (0.12-1.70) | 0.25 |
| Medications |  |  |
| ACEIs or ARBs | 0.82 (0.26-2.70) | 0.73 |
| Beta blockers | 0.60 (0.13-2.14) | 0.45 |
| Diuretics | 1.36 (0.44-4.30) | 0.59 |
| Statins | 0.67 (0.20-2.05) | 0.48 |
| Electrocardiographic data |  |  |
| Intraventricular block | 1.88 (0.37-7.61) | 0.42 |
| PR interval (ms) | 1.01 (0.99-1.03) | 0.27 |
| QRS duration (ms) | 1.03 (1.00-1.06) | 0.03 |
| QTc (ms) | 1.03 (1.01-1.06) | 0.002 |
| RV5 + SV1 voltage (mV) | 1.46 (0.96-2.35) | 0.08 |
| Transthoracic echocardiographic data |  |  |
| AVA (cm^2^) | 1.97 (0.08-45.4) | 0.66 |
| Mean pressure gradient (mmHg) | 0.98 (0.95-1.02) | 0.24 |
| LVMI (g/m^2^) | 1.02 (1.00-1.04) | 0.01 |
| LVEF (%) | 0.93 (0.86-1.00) | 0.06 |
| Severe MR | 1.10e-6 ( -7.47) | 0.37 |
| TR pressure gradient (mmHg) | 0.96 (0.89-1.03) | 0.25 |
| *ACEI* angiotensin-converting enzyme inhibitor, *ARB* angiotensin II receptor blocker, *AVA* aortic valve area, *LVEF* left ventricular ejection fraction, *LVMI* left ventricular mass index, *MR* mitral regurgitation, *NT-proBNP* N-terminal pro-brain natriuretic peptide, *NYHA* New York Heart Association, *QTc* corrected QT interval, *STS* Society of Thoracic Surgeons, *TR* tricuspid regurgitation | | |
